# Predictive value of hemoglobin, serum PAF, and IL-17 in patients with radiation enteritis complicated with intestinal obstruction and construction and validation of predictive model

**DOI:** 10.3389/fmed.2025.1599668

**Published:** 2025-08-01

**Authors:** Meng Wang, Yang Zhao, Wenqiang Ren

**Affiliations:** ^1^Department of Infectious Diseases, Central Hospital Affiliated to Shandong First Medical University, Jinan, China; ^2^Department of Anus and Intestine Surgery, Feicheng People's Hospital, Feicheng, China; ^3^Department of Gastroenterology, Central Hospital Affiliated to Shandong First Medical University, Jinan, China

**Keywords:** radiation enteritis, intestinal obstruction, hemoglobin, platelet activation factor, interleukin-17, prediction model

## Abstract

**Objective:**

To explore the predictive value of hemoglobin, serum platelet-activating factor (PAF), and interleukin-17 (IL-17) for intestinal obstruction in patients with radiation enteritis, and to construct and validate a related prediction model.

**Methods:**

A total of 234 patients who received radiotherapy and were diagnosed with radiation enteritis in our hospital from January 2018 to December 2023 were included in the study. The patients were divided into training set (*n* = 164) and verification set (*n* = 70) according to the ratio of 7:3. The hemoglobin, serum PAF and IL-17 levels of the patients were detected, and the above indicators such as age, gender, radiation dose, radiation site, radiation course, basic diseases (such as hypertension and diabetes), intestinal operation history, chemotherapy history, C-reactive protein(CRP), procalcitonin, albumin, globulin, fibrinogen, D-dimer were collected. The risk factors affecting intestinal obstruction in patients with radiation enteritis were screened by univariate analysis and multivariate Logistic regression analysis, a nomogram prediction model was constructed, the receiver operating characteristic curve (ROC) was drawn, and the calibration curve was used to evaluate the effectiveness of the model. The decision curve analysis (DCA) was used to evaluate the value of clinical application.

**Results:**

Multi-factor Logistic regression analysis showed that diabetes, decreased hemoglobin level, increased serum PAF and IL-17 levels, CRP, were the independent risk factors for intestinal obstruction in patients with radiation enteritis (*p* < 0.05). The constructed nomogram prediction model showed good calibration and fit in the training set and verification set, with C-index of 0.757 and 0.772, respectively, area under ROC curve of 0.759 (95% CI: 0.665–0.853) and 0.775 (95% CI: 0.610–0.939). DCA analysis showed that the model had significant clinical application value within a specific threshold probability range.

**Conclusion:**

The nomogram prediction model constructed by diabetes, hemoglobin, serum PAF, and IL-17 combined with multiple indicators has good prediction efficiency for radiation enteritis patients complicated with intestinal obstruction, which is conducive to identifying high-risk patients in the early clinical stage and taking effective intervention measures.

## 1 Introduction

Radiation enteritis is an intestinal complication caused by radiotherapy for pelvic, abdominal and retroperitoneal malignant tumors, and its incidence rate is gradually rising with the extensive application of radiotherapy in tumor treatment (1). As one of the serious complications of radiation enteritis, it not only increases the suffering of patients and prolongs the hospital stay, but also significantly affects the prognosis and quality of life of patients (2). Early and accurate prediction of the risk of intestinal obstruction in patients with radiation enteritis is crucial for timely intervention and improvement of patient outcomes (3). Hemoglobin, as an important indicator reflecting the anemia status of the body, has important significance in the condition evaluation of many diseases (4). Anemia may affect the oxygen supply to the intestinal tissue, thus affecting the normal repair and function of the intestinal tract, which may be related to the occurrence and development of radiation enteritis and the occurrence of complications (5). Serum platelet-activating factor (PAF) is a phospholipid mediator with extensive biological activity and is involved in the processes of inflammatory response and thrombosis. In radiation enteritis, PAF may promote the occurrence of intestinal obstruction by mediating the inflammatory response and intestinal microcirculation disorders (6). Interleukin-17(IL-17) is a pro-inflammatory cytokine that plays an important role in intestinal inflammatory diseases, and its level change may be related to the pathological process of radiation enteritis complicated with intestinal obstruction. At present, there are few studies on the predictive value of hemoglobin, serum PAF, and IL-17 for complicated intestinal obstruction and related prediction models. The purpose of this study was to explore the predictive value of these indicators, and to construct and validate prediction models, so as to provide a more effective prediction tool for clinical practice and guide clinical decision-making (7).

## 2 Objects and methods

### 2.1 Subjects

A total of 234 patients who received radiation therapy and were diagnosed with radiation enteritis in our hospital from January 2018 to December 2023 were selected. Inclusion criteria: (1) Radiation enteritis confirmed by clinical symptoms, enteroscopy and pathological biopsy; (2) Age ≥18 years old; (3) Patients and their families informed consent and signed informed consent form. Exclusion criteria: (1) Intestinal obstruction caused by combination with other reasons (such as intestinal tumor and volvulus); (2) Suffering from serious liver and renal insufficiency, hematological system diseases, autoimmune diseases and other diseases that can be used for the detection of impact indicators; (3) Intestinal obstruction existed before radiotherapy; (4) patients with incomplete clinical data.

According to the complication of intestinal obstruction, the patients were divided into the group with intestinal obstruction (*n* = 57) and the group without intestinal obstruction (*n* = 107). The diagnosis of complicated intestinal obstruction was based on clinical manifestations (abdominal pain, abdominal distension, vomiting, cessation of exhaust and defecation, etc.), abdominal X-ray, CT and other imaging findings.

### 2.2 Indicator testing

Five milliliters of fasting venous blood was collected in the morning and centrifuged at 3000 rpm for 10 min. Serum was separated. Hemoglobin levels were measured using an automatic biochemical analyzer, and enzyme-linked immunosorbent assay (ELISA) was employed to quantify serum PAF and IL-17 levels. The kits were purchased from Nanjing Saihongrui Biotechnology Co., Ltd. and operated in strict accordance with the kit instructions. Meanwhile, the indicators such as age, gender, radiation dose, radiation site, radiation course, basic disease (hypertension, diabetes, etc.), intestinal surgery history, chemotherapy history, C-reactive protein (CRP), procalcitonin (PCT), albumin, globulin, fibrinogen, and D-dimer were collected.

### 2.3 Establishment of prediction model

The single factor analysis of clinical characteristics was used to screen the possible influencing factors of radiation enteritis patients complicated with intestinal obstruction. Subsequently, variables with *p* < 0.05 in the univariate analysis (e.g., radiotherapy dose, radiotherapy site, diabetes, hemoglobin, serum PAF, IL-17, CRP, fibrinogen, D-dimer) were included in the multivariate Logistic regression analysis. A backward stepwise regression method was applied with a removal criterion of *p* > 0.1, and the variance inflation factor (VIF) was used for multicollinearity diagnosis. Variables with VIF < 10 were retained to construct the final prediction model. The number of events per variable (EPV) was calculated as the number of intestinal obstruction events (*n* = 57) divided by the number of final predictors (5), resulting in an EPV of 11.4, which meets the recommended threshold of ≥10 to minimize overfitting.

### 2.4 Evaluation and validation of prediction model

234 patients were randomly divided into a training set (*n* = 164) and a verification set (*n* = 70). The ROC curve and the calibration curve were drawn in the training set to evaluate the prediction performance of the nomogram model, and they were verified in the verification set. At the same time, decision curve analysis (DCA) was applied to evaluate the clinical application value of the model, so as to assist in making clinical decisions.

### 2.5 Statistical methods

SPSS 26.0 statistical software and R language 4.1.3 software were used for data processing and analysis. The measurement data were expressed as (x̄ s) when they conformed to the normal distribution, and the comparison between two groups was examined by independent sample t test. M (Q1, Q3) was used when the data did not conform to the normal distribution, and Mann–Whitney U test was used for comparison between groups. Enumeration data were compared using *χ* test or Fisher exact probability method. Multivariate Logistic regression analysis was used to screen the risk factors, and the difference with *p* < 0.05 was statistically significant. The nomogram model was established with the software “rms” of R software package, and the working curve of subjects (ROC) drawn with the software “pROC” was used to analyze the prediction value of the model. The Bootstrap method was used to internally verify the model, and the calibration curve of the prediction results and the actual results was drawn. The model consistency index (C-index) was calculated. Hosmer-Lemeshow test was used to evaluate the goodness of fit of the prediction model. The decision curve was drawn using “DCA.r” to analyze the value of the model in clinical application.

## 3 Results

### 3.1 Comparison of general data and indicators of patients between the two groups

In the training cohort of 164 patients, 57 (34.75%) developed intestinal obstruction, while in the validation cohort of 70 patients, 21 (30.00%) presented with this complication. There was no significant difference in general clinical characteristics such as age, gender, and most laboratory indicators between the two groups (*p* > 0.05), as shown in Table 1.

**Table 1:** Comparison of general clinical characteristics of patients between training set and verification set.

Index	Training set (*n* = 164)	Validation set (*n* = 70)	statistical values	*p* value
Age (years)	53.26 ± 8.21	54.13 ± 8.25	0.7412	0.459
Gender (male/female, case)	85/79	38/32	0.118	0.730
Radiotherapy dose ≥50Gy (Yes/No, case)	90/74	35/35	0.469	0.493
The radiotherapy site was the pelvis (Yes/No, case)	110/54	45/25	0.170	0.679
Hypertension (yes/no, case)	40/124	18/52	0.046	0.830
Diabetes (Yes/No, case)	35/129	15/55	0.002	0.988
History of Intestinal Surgery (Yes/No, case)	41/123	18/52	0.013	0.908
Chemotherapy History (Yes/No, case)	60/104	25/45	0.016	0.899
Radiotherapy course (times)	25.54 ± 3.21	26.21 ± 3.25	1.4565	0.146
Hemoglobin (g/L)	115.55 ± 13.41	114.35 ± 12.35	0.646	0.518
Serum PAF (ng/mL)	4.82 ± 1.15	4.63 ± 1.72	0.989	0.323
IL – 17 (pg/mL)	28.35 ± 7.41	27.63 ± 6.71	0.699	0.484
CRP (mg/L)	12.36 ± 4.27	12.45 ± 3.24	0.157	0.874
PCT (ng/mL)	0.22 ± 0.09	0.21 ± 0.08	0.803	0.422
Albumin (g/L)	36.59 ± 3.56	36.13 ± 3.41	0.916	0.360
Globulin (g/L)	31.24 ± 3.62	30.25 ± 3.56	1.925	0.055
Fibrinogen (g/L)	3.82 ± 0.37	3.76 ± 0.62	0.916	0.360
D-dimer (mg/L)	0.63 ± 0.28	0.71 ± 0.32	0.915	0.566

### 3.2 Univariate analysis of radiation enteritis patients complicated with intestinal obstruction

The results of univariate analysis showed that radiation dose, radiation site, diabetes, hemoglobin, serum PAF, IL-17, CRP, fibrinogen, and D-dimer were related to intestinal obstruction in patients with radiation enteritis (*p* < 0.05), as shown in Table 2.

**Table 2:** Univariate analysis of intestinal obstruction in patients with radiation enteritis in training set.

Index	Group complicated with intestinal obstruction (*n* = 57)	Group without complicated intestinal obstruction (*n* = 107)	Statistical values	*p* value
Age (years)	54.52 ± 8.21	55.13 ± 8.25	0.451	0.652
Gender (male/female, case)	30/27	55/52	0.022	0.880
Radiotherapy dose ≥50Gy (Yes/No, case)	40/17	50/57	8.256	0.004
The radiotherapy site was the pelvis (Yes/No, case)	47/10	63/44	9.360	0.002
Hypertension (yes/no, case)	18/39	22/85	2.448	0.117
Diabetes (yes/no, case)	19/38	16/91	7.484	0.006
History of Intestinal Surgery (Yes/No, case)	17/40	24/83	1.084	0.297
Chemotherapy History (Yes/No, case)	21/36	39/68	0.002	0.960
Radiotherapy course (times)	28.59 ± 3.54	27.12 ± 3.78	2.423	0.016
Hemoglobin (g/L)	115.63 ± 10.43	121.32 ± 12.34	2.961	0.003
Serum PAF (ng/mL)	5.51 ± 1.27	4.98 ± 1.03	2.889	0.004
IL-17 (pg/mL)	35.21 ± 8.13	32.54 ± 6.15	2.360	0.019
CRP (mg/L)	15.14 ± 5.32	13.35 ± 3.21	2.685	0.008
PCT (ng/mL)	0.31 ± 0.12	0.29 ± 0.11	1.074	0.284
Albumin (g/L)	36.98 ± 4.56	38.05 ± 3.24	1.740	0.083
Globulin (g/L)	30.52 ± 3.01	31.12 ± 3.35	1.130	0.259
Fibrinogen (g/L)	4.51 ± 0.82	4.23 ± 0.63	2.134	0.016
D-dimer (mg/L)	0.85 ± 0.31	0.72 ± 0.25	2.912	0.004

### 3.3 Multi-factor logistic regression analysis of patients with radiation enteritis complicated with intestinal obstruction

Complicated intestinal obstruction was taken as the dependent variable (0 = none, 1 = yes), and the factor *p* < 0.05 in the single factor analysis was taken as the covariate for multi-factor Logistic regression analysis (see Table 3 for the variable assignment table). The results showed that diabetes, decreased hemoglobin level, increased serum PAF and IL-17 levels, CRP were the independent risk factors for intestinal obstruction in patients with radiation enteritis (*p* < 0.05). In the regression model, the tolerance of each variable was > 0.1, VIF was < 10, and condition index was < 30. In addition, the proportion of variances of multiple covariates without the same feature value was > 50%. Hence, there was no collinearity of each covariate, as shown in Table 4.

**Table 3:** Variable assignment method.

Variable	Meaning	Evaluation
X1	Radiotherapy dose	0 < 50Gy, 1 ≥ 50Gy
X2	The radiotherapy site was the pelvis	0 = none, 1 = yes
X3	diabetes	0 = none, 1 = yes
X4	hemoglobin	continuous variable
X5	Serum PAF	continuous variable
X6	IL-17	continuous variable
X7	CRP	continuous variable
X8	Fibrinogen	continuous variable
X9	D-dimer	continuous variable
Y	Complicated intestinal obstruction	Complicated intestinal obstruction = 1, no intestinal obstruction = 0

**Table 4:** Multivariate analysis of intestinal obstruction in patients with radiation enteritis in training set.

Factor	*B*	Standard error	*Wald*	*p*	*OR*	95% confidence interval
Diabetes	1.266	0.461	7.529	0.006	3.546	1.436–8.759
Hemoglobin	−0.052	0.017	9.135	0.003	0.949	0.917–0.982
Serum PAF	0.462	0.177	6.778	0.009	1.587	1.121–2.247
IL-17	0.058	0.028	4.427	0.035	1.060	1.004–1.119
CRP	0.128	0.046	7.636	0.006	1.137	1.038–1.245

### 3.4 Radiation enteritis patients complicated with intestinal obstruction nomogram prediction model

**Figure 1:**
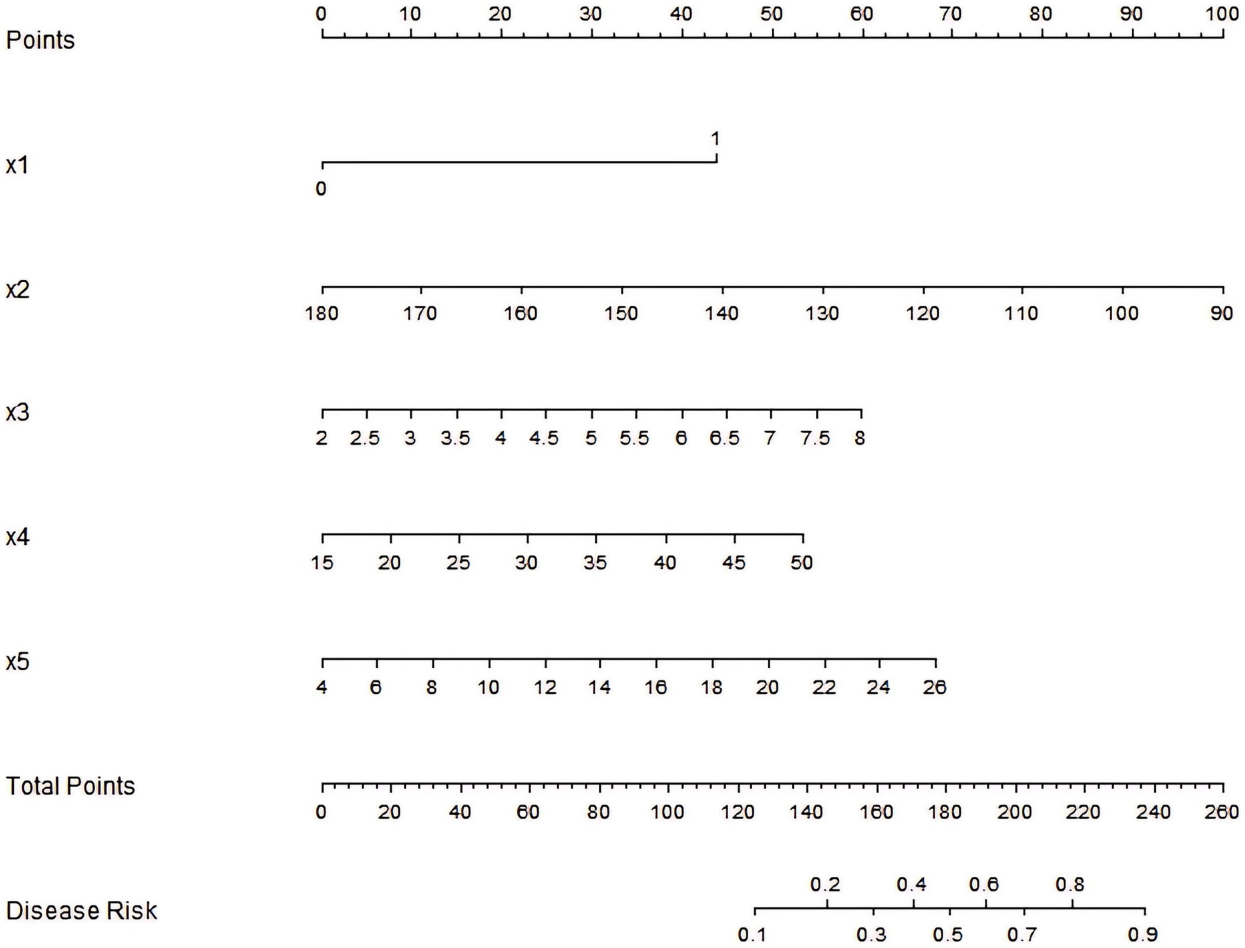
Nomogram prediction model for intestinal obstruction in patients with radiation enteritis. x1: diabetes; x2: hemoglobin; x3: serum PAF; x4: IL-17; x5: CRP.

Based on the independent risk factors identified by multivariate Logistic regression analysis, a nomogram prediction model for radiation enteritis patients complicated with intestinal obstruction was constructed, and the independent risk factors in the model were assigned to calculate the total score for predicting the complicated intestinal obstruction, which was reflected in the probability of predicting the complicated intestinal obstruction. The higher the total score was, the higher the accuracy was in predicting the complicated intestinal obstruction of patients. See Figure 1.

### 3.5 Assessment and validation of nomogram prediction model for radiation enteritis patients complicated with intestinal obstruction

**Figure 2:**
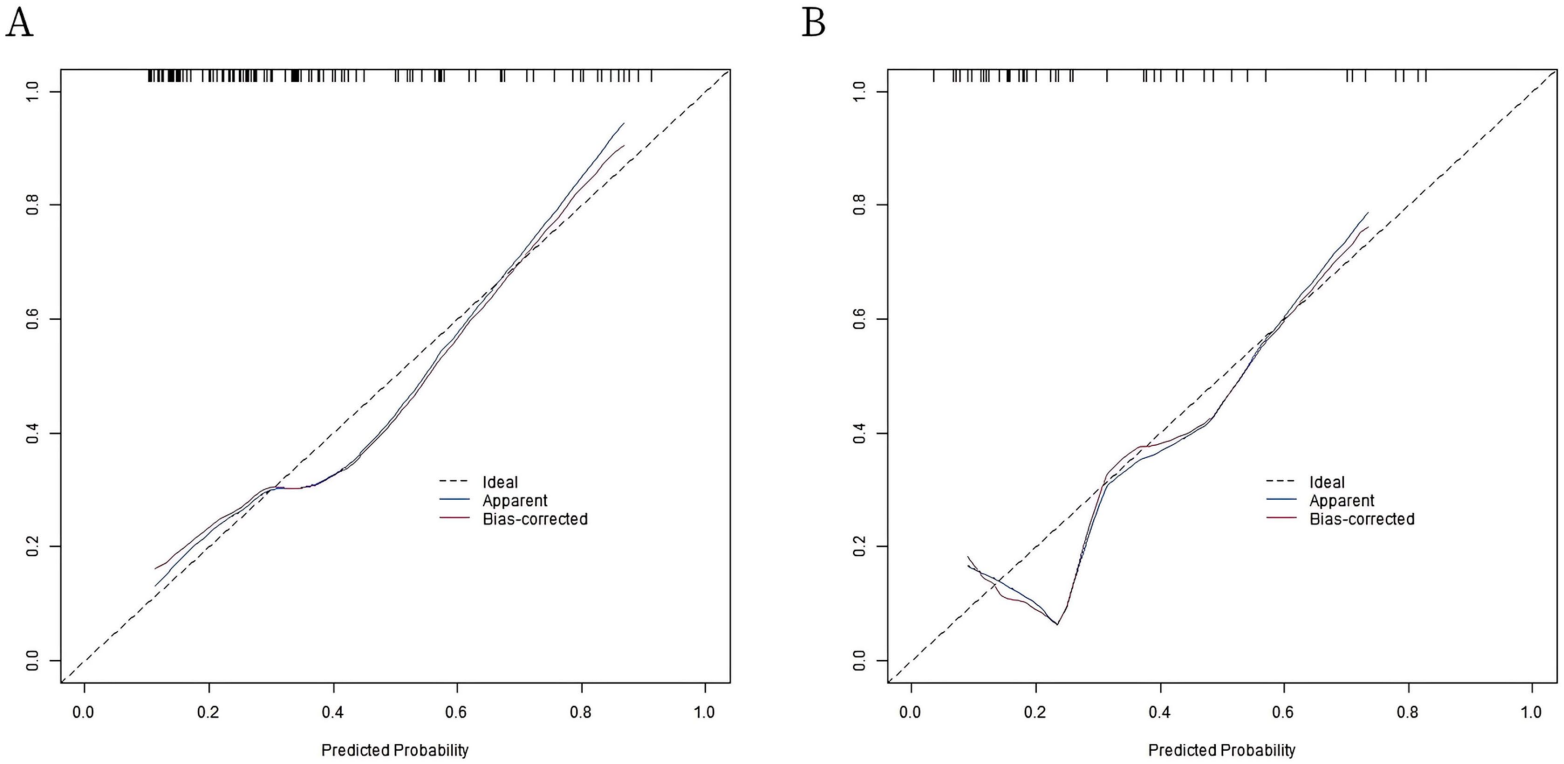
Calibration curves. **(A)** Calibration curve of radiation enteritis combined with intestinal obstruction prediction model in the training set; **(B)** Calibration curve of radiation enteritis combined with intestinal obstruction prediction model in validation set.

**Figure 3:**
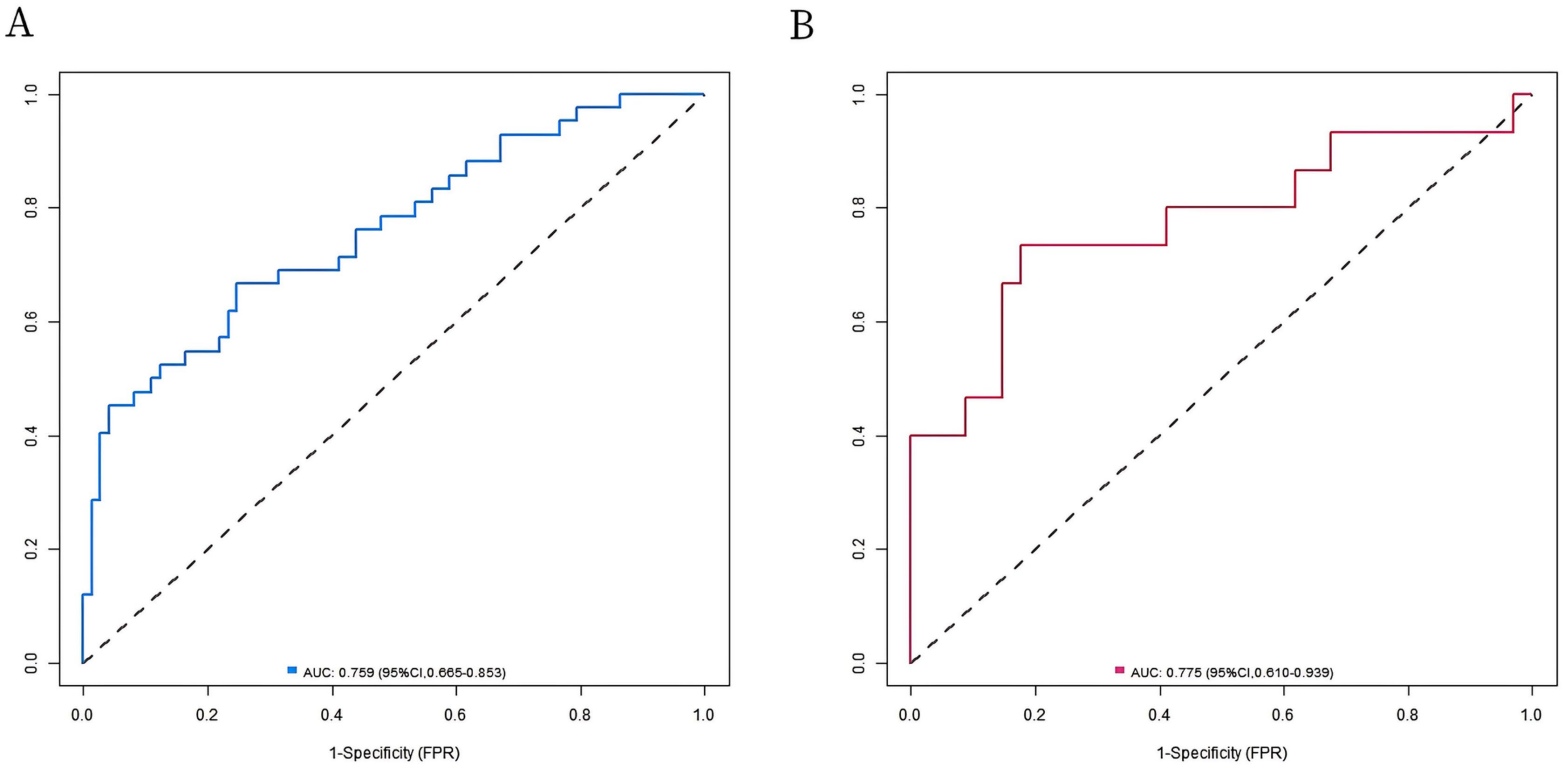
Receiver operating characteristic (ROC) curves. **(A)** ROC curve of radiation enteritis combined with intestinal obstruction prediction model in the training set; **(B)** ROC curve of radiation enteritis combined with intestinal obstruction prediction model in validation set.

In the training set and verification set, the C-index values of the nomogram models were 0.757 and 0.772, respectively. The calibration curve showed that the predicted value accorded well with the true value. The results of Hosmer-Lemeshow test were χ^2^ = 6.180, *p* = 0.627 and χ^2^ = 6.597, *p* = 0.581, respectively. The ROC curves were displayed in the training set and the verification set, and the AUC of the nomogram model for intestinal obstruction in patients with radiation enteritis was 0.759 (95% CI: 0.665–0.853) and 0.775 (95% CI: 0.610–0.939), respectively, and the sensitivity and specificity were 0.667, 0.753 and 0.733, 0.588, respectively. The calibration curve is shown in Figure 2 and the ROC curve is shown in Figure 3.

### 3.6 Radioactive enteritis patients complicated with intestinal obstruction nomogram prediction model of decision curve analysis

**Figure 4:**
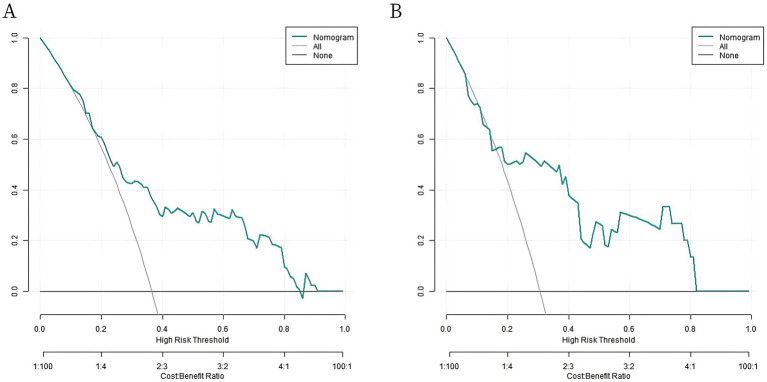
Decision curves. **(A)** Decision curve of radiation enteritis combined with intestinal obstruction prediction model in the training set; **(B)** Decision curve of radiation enteritis combined with intestinal obstruction prediction model in validation set.

Analysis of decision curve showed that when the threshold probability was between 0.08 and 0.95, the application of the nomogram model constructed in this study to predict intestinal obstruction in patients with radiation enteritis had more clinical benefits than the preoperative decision that all patients would relapse or all patients would not relapse, as shown in Figure 4.

## 4 Discussion

Radiation enteritis complicated with intestinal obstruction seriously affects the prognosis of patients, and accurate prediction of its occurrence is of great significance for clinical treatment (8). In this study, we constructed the nomogram prediction model based on multiple indicators, which provided a new tool for clinical evaluation. However, the study has certain limitations, and the absence of external verification is an important aspect that needs to be further discussed (9).

From the results of this study, multivariate Logistic regression analysis identified diabetes, decreased hemoglobin levels, increased serum PAF and IL-17 levels, CRP as independent risk factors for intestinal obstruction in patients with radiation enteritis. Diabetes is a common systemic disease, and its resulting microangiopathy and neuropathy may affect the normal physiological function of the intestinal tract (10). Hyperglycemia can cause damage to the intestinal vascular endothelial cells and increase the blood viscosity, affecting the blood supply to the intestinal tract, leading to hypoxia and ischemia of the intestinal tissue, and further affecting the repair and regeneration ability of the intestinal tract. At the same time, diabetic neuropathy can cause intestinal peristalsis dysfunction, make the intestinal content transmission is not free, increase the risk of intestinal obstruction (11).

The decreased hemoglobin level reflects the possible anemia in the patient, which leads to insufficient oxygen supply to intestinal tissues and affects the normal metabolism and repair of intestinal cells. Intestinal mucosa needs sufficient oxygen for repair and regeneration after radioactive damage. However, anemia weakens this process and makes the intestinal tract more vulnerable to pathological changes such as adhesion and stenosis, which will eventually lead to the (12). Serum PAF and IL-17 were considered as inflammation-related factors, and their elevated levels indicated that the body was in an inflammatory activation state. PAF has a wide range of biological activities, which can promote platelet aggregation, increased vascular permeability and inflammatory cell infiltration, leading to the aggravation of the reaction (13). IL-17 can induce the production of a variety of cytokines and chemokines, further amplify the inflammatory response, damage the intestinal tissue, promote the process of fibrosis, and increase the risk of intestinal obstruction (14).

CRP, a sensitive marker of the inflammatory response, plays a crucial role in the pathogenesis of radiation enteritis complicated with intestinal obstruction. Elevated CRP levels reflect the intensity of systemic and local inflammatory reactions, which are central to the progression of radiation-induced intestinal injury (15). In this study, CRP was identified as an independent risk factor for intestinal obstruction (OR = 1.137, 95% CI: 1.038–1.245), indicating that each 1 mg/L increase in CRP is associated with a 13.7% higher risk of obstruction. This finding aligns with previous research showing that CRP correlates with the severity of intestinal inflammation and fibrosis in radiation enteritis. Clinically, monitoring CRP levels alongside the nomogram model can enhance risk stratification. Patients with both high CRP (>15 mg/L) and a nomogram score ≥65 demonstrate a particularly high risk of obstruction, warranting aggressive intervention strategies (16).

The nomogram prediction model constructed in this study showed good calibration and fitting in the training set and the verification set, and indicators such as C-index index and the area under the ROC curve showed that the model had certain prediction performance. The nomogram prediction model constructed in this study, which incorporates independent risk factors such as diabetes, hemoglobin, serum PAF, IL-17, and CRP, can be integrated into clinical practice through a structured workflow. Clinicians can input patients’ baseline data (e.g., laboratory indices, medical history) into the model to calculate a total risk score, which directly correlates with the probability of developing intestinal obstruction in patients with radiation enteritis. This process can be operationalized via electronic health record (EHR) systems by embedding the model as a decision support tool, enabling real-time risk assessment during patient visits or hospital admissions. Additionally, a printable nomogram chart (as shown in Figure 1) can be provided to clinical departments for manual score calculation, ensuring accessibility in settings with limited digital infrastructure. To enhance clinical utility, the model’s risk stratification should be categorized into three tiers: low-risk (probability < 30%), intermediate-risk (30–60%), and high-risk (probability > 60%). This stratification allows for personalized management strategies, as outlined below. Threshold Scores and Intervention Triggers, high-risk patients (score ≥ 70 points, corresponding to a probability > 60%): Immediate intensive intervention is warranted, including: enhanced surveillance: Weekly monitoring of hemoglobin, CRP, and inflammatory markers (PAF, IL-17) to track disease progression. Proactive nutrition support: Referral to a dietitian for high-protein, iron-rich diets or iron supplementation to address anemia, combined with anti-inflammatory nutritional interventions (e.g., omega-3 fatty acids). Early radiological assessment: Scheduled abdominal CT scans every 1–2 months to detect early signs of intestinal stenosis or adhesion. Multidisciplinary consultation: Collaboration with gastroenterologists and surgeons to develop preventive strategies (e.g., dietary modification, probiotic therapy to regulate intestinal microbiota). Intermediate-risk patients (score 40–69 points, probability 30–60%): Moderate intervention focused on risk mitigation: monthly laboratory follow-ups: Monitoring of hemoglobin and inflammatory markers to detect trends. Lifestyle modification: Counseling on fluid intake, fiber optimization, and regular physical activity to promote intestinal motility. Educational interventions: Patient education on recognizing early signs of obstruction (abdominal pain, vomiting) to facilitate timely seeking of medical attention. Low-risk patients (score < 40 points, probability < 30%): Routine care with periodic screening: quarterly laboratory checks: Basic monitoring of hemoglobin and CRP to ensure stability. Standard follow-up: routine clinical visits without intensive interventions, with emphasis on maintaining overall health.

Impact on Patient Management, early risk identification: the model enables proactive rather than reactive management, potentially reducing the incidence of severe obstruction and associated hospitalizations. For example, high-risk patients can be prioritized for preventive measures (e.g., strict glycemic control for diabetic patients) to mitigate microvascular damage and inflammatory responses. Resource optimization: by stratifying patients into risk tiers, healthcare resources can be allocated efficiently. High-risk cases receive intensive monitoring, while low-risk cases require minimal intervention, reducing unnecessary testing and costs. Personalized treatment plans: the model supports tailoring interventions to individual patient profiles. For instance, patients with low hemoglobin may receive iron therapy, while those with elevated PAF/IL-17 could be considered for anti-inflammatory therapies (e.g., corticosteroids under close supervision). Improved patient outcomes: early intervention in high-risk patients may delay or prevent intestinal obstruction, thereby reducing morbidity, improving quality of life, and potentially decreasing mortality associated with severe complications.

However, the absence of external validation is an important limitation of this study. External validation was not performed primarily for the following reasons: First, there is currently a lack of open datasets that highly match the data structure and patient characteristics of this study. Clinical data of patients with radiation enteritis have strong heterogeneity, and patients in different regions and different hospitals have differences in radiation therapy plan, the distribution of basic diseases, treatment compliance, etc. (17). Using mismatched external data for validation may not accurately assess model performance or even lead to erroneous conclusions. Second, there are many practical difficulties in obtaining external data for verification. Multi-center cooperation needs to coordinate ethical approval and data sharing policies among different hospitals, which is a complex and time-consuming process. Furthermore, data collection standards and test methods may differ among hospitals, which may also affect the accuracy of verification results. In practice, it is difficult to ensure that the quality and consistency of external data are the same as data from this study.

Although the model in this study performed well in internal validation, the lack of external validation may hinder its promotion and application in different clinical environments. External validation is crucial to test the stability and generalization ability of the model, i.e., whether the model can still maintain good prediction performance in different patient groups and medical settings (18). For example, radiotherapy equipment, techniques, and treatment strategies for radiation enteritis may vary across hospitals, which could lead to different clinical outcomes for patients and thus affect the prediction accuracy of the model (19). Therefore, future research should actively seek multi-center cooperation, obtain more external data for validation, and incorporate potential influencing factors such as gene polymorphism and lifestyle factors (e.g., diet and exercise habits) to improve the reliability and applicability of the model.

In addition, there are some other limitations in this study. The relatively small sample size may affect the stability and reliability of the results to some extent. A small sample size may not cover all possible patient characteristics and clinical conditions, which leads to a certain deviation of the predictive efficiency of the model. In the follow-up research, we should enlarge the sample size and conduct large sample, multi-center research to improve the accuracy and universality of the model. At the same time, only some factors that might affect the intestinal obstruction in patients with radiation enteritis were included in this study, and some potential confounding factors such as gene polymorphism and factors (such as diet and exercise habits) were not considered (20). Gene polymorphism may affect individual susceptibility to radiation injury and the intensity of inflammatory response. Lifestyle factors may also indirectly affect the occurrence, development and complications of radiation enteritis by affecting the intestinal microecology and the body immunity (21). Future research can further incorporate these factors, improve the prediction model, and improve its prediction ability.

In summary, the nomogram prediction model based on multiple indicators such as diabetes, hemoglobin, serum PAF, and IL-17 constructed in this study has certain prediction efficiency for intestinal obstruction in patients with radiation enteritis. However, the lack of external validation limits the model’s generalizability in different clinical settings. Future studies are needed to overcome practical difficulties, carry out multi-center, large-sample research, incorporate more potential influencing factors (such as gene polymorphism and lifestyle factors), and conduct external validation to further improve the prediction model, so as to provide more powerful support for early clinical identification of high-risk patients and the formulation of personalized treatment strategies.

## Data Availability

The raw data supporting the conclusions of this article will be made available by the authors, without undue reservation.
